# What Are the Correlates of Global Variations in the Prevalence of Opioid Use Disorders? An Analysis of Data From the Global Burden of Disease Study, 2019

**DOI:** 10.7759/cureus.18758

**Published:** 2021-10-13

**Authors:** Ravi P Rajkumar

**Affiliations:** 1 Psychiatry, Jawaharlal Institute of Postgraduate Medical Education and Research, Pondicherry, IND

**Keywords:** religion, unemployment, depression, culture, epidemiology, opioid use disorder, opioid epidemic

## Abstract

Introduction

The recent opioid crisis in North America has brought the problem of opioid use disorders (OUD) into clinical and public health focus, with experts warning that other countries or regions may be at future risk of experiencing such crises. The existing literature suggests that a wide range of social, cultural and economic factors may be associated with the onset, course and outcome of OUD in individuals. The current study uses data on the estimated prevalence of OUDs across 115 countries, obtained from the Global Burden of Disease Study, 2019, to examine the bivariate and multivariate associations between national prevalence of OUD and these factors.

Methods

Data on the estimated prevalence of OUDs was obtained via a database query from the Global Burden of Disease (GBD) Collaborative Network database for the year 2019. Recent (2018-2019) data on 10 relevant variables identified in the literature (gross national income, economic inequality, urbanization, social capital, religious affiliation and practice, unemployment, divorce, cultural individualism, and prevalence of depression) were obtained from the GBD, World Bank and Our World in Data databases. After transformation to a normal distribution, bivariate and univariate analyses were conducted to identify the significance and strength of the associations between these variables and the prevalence of OUD.

Results

Of the 10 variables studied, all variables except the divorce rate and religious affiliation were significantly correlated with the prevalence of OUD on bivariate analyses, though the strength of these associations was in the poor to fair range. On multivariate analysis, a significant association was observed only for the prevalence of depression, with trends towards a positive association for cultural individualism and unemployment, and a protective trend observed for religious practice.

Discussion

Though subject to certain limitations inherent in cross-sectional analyses, these results suggest that certain variables may be associated with a higher prevalence of OUD at the national level. Replication and refinement of these analyses may prove useful in identifying countries or regions at risk of a future opioid epidemic or crisis, which could facilitate the institution of preventive measures or early intervention strategies.

## Introduction

The ongoing opioid crisis affecting the United States of America has brought the study of opioid use disorders into focus in recent years. This crisis has been associated with numerous local and regional “outbreaks” or “hot spots” of increased use, as well as significant increases in the incidence of adverse health outcomes such as overdoses and blood-borne infectious diseases [1]. However, opioid use disorders are a global health problem of significant scope, and are endemic in many other regions of the world. In an analysis of data from the Global Burden of Disease 2010 Study, it was estimated that opioid dependence accounted for over 9 million disability-adjusted life years (DALYs) globally, or 0.4% of all global DALYs; besides North America, Eastern Europe and parts of sub-Saharan Africa were found to be more severely affected than other regions. It was also observed that there was an increase in opioid use-related DALYs of over 70% in the period 1990-2010 [2]. A re-examination of the global data in 2017 found that these trends were maintained, with over 40 million people estimated to have opioid dependence globally, and a further increase in opioid consumption observed in several European countries [3,4]. Given these figures, some experts have pointed out that it is incorrect to think of the opioid crisis as a regional problem confined to the United States; rates of opioid use disorders continue to rise in several countries, and the situation must be more properly viewed as a global health challenge, particularly in countries where the lack of quality treatment or punitive laws act as barriers to effective care [4,5]. In particular, strict policies related to criminalization of opioid possession or incarceration for the same can lead to stigmatization and delays in seeking treatment [6,7].

At a biological level, opioid use disorders can be studied in terms of the effects of opioids on specific endogenous receptors, with resultant alterations in the functioning of specific brain circuits and regions in a stage-specific manner, leading to initial salience and habit formation, followed by withdrawal and stress sensitivity and finally by deficits in executive functioning [8]. However, in real-life settings, opioid dependence is a complex problem that involves the interaction of biological risk (the diathesis) with social and environmental factors (stressors), and treatment outcomes are optimal only if the latter are sufficiently taken into account [9]. Social factors that have been found to significantly influence the development and outcome of opioid use disorders include poverty and economic inequality, unemployment, levels of urban and rural development, the extent and quality of social support networks, religious or spiritual involvement, and cultural values and practices [10-16]. These factors affect influence individual-level variables such as affective responses, self-image and self-efficacy, social status, loneliness, coping skills and attitudes towards drug use, thereby altering a given person’s likelihood of engaging in or continuing to use opioids [17-19]. It is therefore plausible that some of the observed cross-national or cross-regional variation in opioid use disorders may be due to one or more of these social variables. Examining these associations may be of significant importance in identifying countries or regions that are at future risk of opioid “crises” or “epidemics”, and instituting preventive or early treatment strategies, though such findings may not be applicable to individual cases. This is particularly important in low- or middle-income countries with less-developed health infrastructure, and which may be overwhelmed by a full-blown opioid crisis [19,20].

The current study was carried out with the following aims in mind:

1) To identify social, economic and cultural variables that are significantly associated with the estimated national prevalence of opioid use disorders;

2) To confirm the strength and stability of these associations through multivariate analyses.

## Materials and methods

The current study aimed to examine the association between the most recent estimates for the nation-wise prevalence of opioid use disorders (OUD), based on the Global Burden of Disease 2019 study, and the various social and cultural factors identified as relevant to this group of disorders.

Data sources

Information on the country-wise prevalence of opioid use disorders was obtained through a database query from the Global Burden of Disease Collaborative Network, which provides estimates of this variable for the year 2019 as a percentage [21].

In selecting relevant variables for correlation analyses, two criteria were applied: (a) evidence of a relationship between the variable and the incidence or outcome of OUD in prior research, and (b) availability of a reliable and valid data source for the variable. A literature search of the PubMed and EMBASE database was carried out using the key words “opioid dependence” or “opioid use disorder” along with “demographic”, “socioeconomic”, “economic”, “cultural”, “religious” and “spiritual” (or variations such as “relig*” or “cultur*”) and “prevalence” or “relapse”. A total of 208 citations were retrieved using this method, of which 19 papers provided information on 10 variables which fulfilled the above two criteria. The 10 parameters included in this study, based on these publications, were: gross national income, economic inequality, urbanization, social capital, religious affiliation and practice, unemployment, divorce, cultural individualism, and prevalence of depression. The operational definition for each of the variables included in this analysis, the rationale for their inclusion based on papers retrieved from the literature, and the data source for each variable are summarized in Table 1. Complete information on at least five of the 10 study variables was available for 115 countries; these countries were included in the final analysis. A complete data sheet for this study is available from the author on reasonable request.

**Table 1:** Independent variables analyzed for their association with the national prevalence of opioid use disorder

Variable and method of estimation	Rationale for inclusion	Data source
Gross national income (Atlas method)	Evidence of an association between poverty and opioid use, poorer treatment outcomes and limited access to treatment [10,22,23].	World Bank database [24].
Economic inequality (Gini coefficient)	Socioeconomic inequality interacts with other variables to influence opioid use, and also leads to unequal access to care and poorer outcomes [25,26].	World Bank database [24].
Urbanization (percentage of population living in urban areas)	Rural areas may face a disproportionate burden of opioid misuse in developing countries, while the opposite may be true in developing countries [27,28].	World Bank database [24].
Social capital (Social capital sub-score of the Legatum Prosperity Index)	Social support and cohesiveness are protective against opioid misuse and predict better outcomes in those affected [29,30].	World Bank database [24].
Religious affiliation (percentage of population sample)	Religious affiliation may have a protective effect on the initiation and maintenance of opioid use [14].	Pew Research Center report [31].
Religious practice (mean percentage of population sample reporting engagement in prayer, religious rituals, or considering religion important in their lives)	Frequent religious attendance / practice is associated with lower rates of substance use, even in vulnerable or deprived populations [32].	Pew Research Center report [31].
Unemployment (percentage of work force currently not in employment)	At a regional level, increases in unemployment rate have been correlated with several indices of opioid misuse [33].	World Bank database [24].
Divorce (crude divorce rate per 1000 population)	Marital separation is significantly associated with opioid misuse and dependence [34,35].	Our World In Data database [36].
Cultural individualism (Hofstede’s index of individualism / collectivism)	Among cultural variables, only individualism / collectivism has been associated with the risk of opioid misuse [37,38].	Hofstede Institute database [39].
Depression (prevalence %)	Opioid use and misuse have been consistently associated with major depression in all age groups [40,41,42]. This association was not observed for other common mental disorders [42].	Global Burden of Disease 2019 Collaborative Network database [21].

Data analysis

Data analysis was carried out in two stages: bivariate and multivariate analyses. Prior to data analysis, all study variables were tested for normality using the Shapiro-Wilk test. As they all showed significant deviations from normality, they were converted to an approximately Gaussian distribution using a natural logarithmic transformation.

For the purposes of bivariate analyses, correlations between the estimated prevalence of OUD and the 10 independent risk/protective factors identified in the literature were assessed using Pearson’s correlation coefficient (r). All analyses were two-tailed, and a significance level of p < .05 was considered to represent a meaningful correlation. To verify the independence of the 10 study variables, correlation coefficients between them were computed; a Pearson’s r of 0.8 or greater was taken as indicating significant multicollinearity. The strength of observed correlations was graded as poor (0.1 < r < 0.3), fair (0.3 < r < 0.6), moderate (0.6 < r < 0.8) or strong (r > 0.8) based on standard guidelines for medical research [43].

To confirm the strength and reliability of these associations, a multivariate linear regression analysis was carried out using the backward regression method, with the prevalence of OUD as the dependent (outcome) variable. In this approach, all independent variables significantly associated with the prevalence of opioid use disorders in bivariate analysis (threshold p < .05) were entered into the initial model, and variables that were not significantly associated with the outcome were removed from the model step-wise, with only those variables showing a significant association retained in the final model. To ensure that these results were not confounded by multicollinearity, variance inflation factors (VIFs) were computed for each independent variable; a VIF greater than 4 was taken as indicating possible multicollinearity, and a VIF > 10 as indicating significant multicollinearity that would interfere with the validity of the regression model.

## Results

Data on a total of 115 countries was analyzed. The results of bivariate correlation analyses are presented in Table 2. The estimated prevalence of OUD was significantly correlated with eight of the 10 independent variables examined in this study. Positive correlations were observed for gross national income (r = .45, p < .001), urbanization (r = .42, p < .001), social capital (r = .26, p = .009), unemployment (r = .21, p = .026), cultural individualism (r = .44, p < .001) and prevalence of depression (r = .50, p < .001), while negative correlations were observed for economic inequality (r = -.25, p = .009) and religious practice (r = -.34, p = .001). No correlation was observed for religious affiliation; while a positive trend was observed for the divorce rate, this did not reach the threshold for significance (r = .19, p = .069). Several of the independent variables showed significant correlations with each other; however, none of these correlations attained the threshold of r > 0.8, suggesting that there was no significant multicollinearity. The strength of these associations ranged from poor to fair.

**Table 2:** Correlation matrix of variables associated with the estimated prevalence of opioid use disorder Abbreviations: Var, variable; OUD, estimated prevalence of opioid use disorder; GNI, gross national income per capita; Gini, Gini coefficient of economic inequality; Urb, % of population residing in urban areas; SC, Legatum index of social capital; RA, % of population affiliated to any religion; RP, % of population reporting regular religious practices; Unemp, % of current labor force unemployed; Div, crude divorce rate; IC, Hofstede index of cultural individualism-collectivism; Dep, estimated prevalence (%) of depressive disorder

Var	1 - OUD	2 - GNI	3 - Gini	4 - Urb	5 - SC	6 - RA	7 - RP	8 - Unemp	9 - Div	10 - IC	11 - Dep
1	*	-.45 (.001)	-.25 (.009)	.42 (.001)	.26 (.009)	-.14 (.187)	-.34 (.001)	.21 (.026)	.19 (.069)	.44 (.001)	.50 (.001)
2	-	*	-.39 (.001)	.73 (.001)	.57 (.001)	-.41 (.001)	-.67 (.001)	-.04 (.684)	.36 (.001)	.55 (.001)	.27 (.003)
3	-	-	*	-.11 (.269)	-.08 (.462)	.22 (.038)	.47 (.001)	.26 (.006)	-.38 (.001)	-.33 (.001)	-.12 (.197)
4	-	-	-	*	.25 (.012)	-.22 (.044)	-.40 (.001)	.27 (.004)	.21 (.053)	.30 (.001)	.32 (.001)
5	-	-	-	-	*	-.16 (.147)	-.20 (.065)	-.22 (.021)	.05 (.686)	.39 (.001)	.05 (.636)
6	-	-	-	-	-	*	.67 (.001)	.29 (.007)	-.17 (.151)	-.26 (.016)	.11 (.321)
7	-	-	-	-	-	-	*	.16 (.133)	-.45 (.001)	-.52 (.001)	-.15 (.179)
8	-	-	-	-	-	-	-	*	-.16 (.145)	.05 (.635)	.29 (.002)
9	-	-	-	-	-	-	-	-	*	.44 (.001)	.31 (.003)
10	-	-	-	-	-	-	-	-	-	*	.37 (.001)

Variables listed as showing significant correlations above were included in the backward multivariate linear regression analysis, the results of which are summarized in Table 3. The final model, arrived at after five steps, was significant overall (F = 12.92, p < .001) and explained approximately 37% of the variation in national prevalence of OUD (R2 = 0.40; adjusted R2 = 0.37). In this model, a significant positive association with opioid use was identified for the prevalence of depression (β = .33, p = .002), while positive trends were identified for unemployment (β = .17, p = .093) and cultural individualism (β = .22, p = .056). A possible protective effect of religious practice was noted (β = -0.20, p = .066), though this failed to reach statistical significance. Variance inflation factors were <4 for all study variables, ruling out significant multicollinearity.

**Table 3:** Multivariate backward linear regression analysis of variables associated with the prevalence of opioid dependence

Variable	Correlation coefficient (β)	Part correlation	Significance level	Variance inflation factor
Depression, prevalence, %	.34	.28	.002	1.43
Hofstede’s index of individualism / collectivism	.22	.17	.056	1.68
Religious practice, % of national sample population	-.20	-.16	.066	1.47
Unemployment, % of total labour force	.17	.15	.093	1.23

As depression may itself be influenced by factors such as unemployment, poverty, cultural values and religiosity, the analysis was repeated with the exclusion of depression; in this secondary analysis, a positive link with opioid use disorders was observed for cultural individualism (β = .40, p < .001) with positive trends for unemployment (β = .18, p = .081) and urbanization (β = .19, p = .086).

## Discussion

The current study aimed to identify associations between specific indicators of social and economic well-being and the estimated prevalence of OUD across countries. Though the indicators included in this study were identified through research in individuals at the community or hospital level, eight of them were also observed to be significant at the national level. This suggests a certain degree of coherence between group-level and individual-level risk factors, though one cannot extrapolate from the current findings to make individual-level recommendations.

It was observed - somewhat counter-intuitively - that a higher gross national income, lower economic inequality, and higher levels of social capital were positively correlated with the prevalence of OUD in bivariate analyses. This is in contrast to individual-level findings where poverty, inequity and lack of social support are risk factors for both OUD and its consequences. However, these results are consistent with the existing epidemiological literature in which high prevalence rates for OUD have been observed in economically developed countries, particularly in North American and European countries [2-4]. None of these variables remained significantly associated with OUD on multivariate analysis, suggesting that they might represent chance or coincidental associations. Another possibility is that factors such as insufficient regulation of prescription opioids, over-prescription of high-potency opioids, and open supply chains for illicit opioid products may be more common in certain regions of the developed world [44-46]. It has also been observed that daily opioid use was positively correlated with social capital in rural residents of the United States of American suggesting that in some communities, drug use may act as a form of “social currency” [47]; however, these associations require replication in settings outside North America.

The single variable most significantly associated with the prevalence of OUD, even after correcting for other variables in multivariate analysis, was the estimated prevalence of depression. This finding is consistent with the conceptualization of the opioid crisis as being related to “despair” [12], with research in individuals showing that significant depressive symptoms are a robust predictor of opioid misuse [40-42], and with the bidirectional association between depression and opioid misuse [48]. At a more fundamental level, there appear to be genetic and neurobiological links between depression and opioid dependence [49,50]. It is perhaps of note that many of the independent variables examined in this study, such as urbanization, unemployment, and cultural individualism, were significantly associated with the prevalence of depression both in prior studies and in the current bivariate analyses (Table 1) [51-53]. This suggests that these variables may influence OUD prevalence at least partly through their effects on depression.

Religious practice, but not religious affiliation, was observed to have a potential “protective” effect - in terms of a negative correlation with the prevalence of OUD - in bivariate analyses. Though this variable was retained in the final multivariate regression model, the association was weakened to a trend level. Nevertheless, this suggests a potential protective effect for religious practices such as prayer and attendance at religious services, rather than for self-reported religious group affiliation, which is consistent with the existing literature in individuals [14,33,54,55]. This finding requires replication using more sensitive methods, and the exact mechanisms underlying this association are unclear.

The other two variables retained in the final regression model were cultural individualism and unemployment. Cultural individualism represents the tendency of a culture to value the individual’s rights against the group, as opposed to collectivism which privileges group values and well-being [56]. High scores on cultural individualism have been associated with certain addiction-related behaviours, including a greater likelihood to consume alcohol for positive reinforcement [57] and greater opioid use in adolescents [38]; on the other hand, cultural collectivism has been associated with social media addiction [58]. It is therefore possible that cultural factors may influence the form of addiction chosen by vulnerable individuals, with self-administration of reinforcing substances being commoner in individualistic cultures, and behavioural addictions involving connectedness or “belonging” being more common in collectivistic cultures, though this association requires careful verification.

Unemployment has been identified as a consistent risk factor for opioid misuse in various community-based samples [33,59-61]. Similar effects have been observed in experimental settings, where patients with OUD who were given an “employment” task had reduced drug-seeking behaviour [62]. A lack of opportunities for employment leads to difficulties in reintegration into one’s community, even after successful detoxification, and is associated with a high risk of relapse [63]. Similar correlations to those reported in this study have been demonstrated at a cross-national level for other substances, suggesting that unemployment is a general risk factor for substance use disorders [64]. Therefore, a high or rising unemployment rate may be a risk factor for increased opioid use in a given country or region, and may even trigger an “opioid crisis” in the presence of other vulnerabilities.

These results are subject to certain important limitations. First, they are based on analyses of cross-sectional data collected at single time points, and no definite inference can be drawn regarding causality. Second, the findings of this study apply to country-level estimates of the prevalence of OUD, and cannot be directly applied to individuals suffering from these disorders. Third, there may be a certain degree of inaccuracy in study variables, as they are based on survey data and/or statistical modelling which are subject to bias or uncertainty due to sampling methods or estimation formulae. Fourth, it was not possible to take into effect the influence of other social factors, such as local legislation or actions by law enforcement, as these could not be easily operationalized. Fifth, these results do not take into account the importance of geographical factors in opioid use disorders, particularly proximity to regions actively involved in the manufacture and distribution of illicit opioids [65]. Sixth, there may be other social and cultural factors that could increase or decrease the risk of opioid misuse, but which have not yet been identified in the literature and were therefore unwittingly omitted in this study. Finally, though this method of cross-national analysis has been used for other substances such as alcohol [59], it has not been applied to the problem of opioid use and dependence so far.

## Conclusions

Despite the limitations listed above, the current study suggests that specific variables - namely, the prevalence of depression, cultural values, religious practice and unemployment rates - are significantly associated with global variations in the prevalence of OUD. Given the warnings from public health experts about looming opioid crises in countries outside North America, these findings may help to identify regions that are at a higher risk of such crises. With further research and refinement of statistical models, it may be possible to achieve greater predictive power - for example, to assess if the combination of cultural individualism, high unemployment, low religiosity and high rates of major depression could identify countries at future risk of an opioid epidemic. This would permit, in principle, the initiation of appropriate and multifaceted preventive or early intervention strategies in terms of legislation, economic stimulus measures, development of appropriate medical infrastructure to treat those affected, and strengthening local religious and community support networks.
